# Sustaining an HIV Prevention and Wellness Program for Sexual Gender Minorities during the COVID-19 Pandemic

**DOI:** 10.3390/ijerph19042114

**Published:** 2022-02-13

**Authors:** Latrice C. Pichon, Megan L. Wilkins, Gisela Guerrero, Andrea L. Williams Stubbs, Edward D. Wiley, Justin Dodson, Carla London, Michelle Teti

**Affiliations:** 1 School of Public Health, The University of Memphis, Memphis, TN 38152, USA; gisela.guerrero@memphis.edu (G.G.); lwllms26@memphis.edu (A.L.W.S.); 2 Department of Infectious Diseases, St. Jude Children’s Research Hospital, Memphis, TN 38105, USA; megan.wilkins@stjude.org; 3 The Memphis Headliners, Memphis, TN 38103, USA; edwiley06@gmail.com (E.D.W.); navigatingcourage@gmail.com (J.D.); carlafnp1@gmail.com (C.L.); 4 Department of Public Health, The University of Missouri, Columbia, IN 65211, USA; tetim@health.missouri.edu

**Keywords:** HIV, social determinants, MSM, LGBTQ+

## Abstract

Improving mental health, body image, and financial stability is paramount to achieving viral suppression and maintaining HIV-negative status for minoritized communities. The purpose of this paper is to describe the lessons learned from maintenance of an HIV prevention and wellness program during the COVID-19 pandemic. A three-session program was implemented in a hybrid format to account for county-wide restrictions and reopening processes. Lessons learned include the utility of a hybrid format, importance of CBPR partnership, innovation in virtual platform, value of social media presence and upkeep, and use of multiple methods to ascertain evaluative data. Sustaining an HIV prevention and wellness program requires strong research collaborations and ongoing engagement with priority populations and the flexibility to pivot as needed.

## 1. Introduction

Several unmet needs have been recognized in Black men who have sex with men (MSM) and Black transgender women living in the Deep U.S. South (subset of nine states) which contribute to high disease rates, poor health outcomes, and socioeconomic challenges [1,2]. Gallup polls suggest a population rate of 3% for individuals who identify as lesbian, gay, bisexual, transgender, and queer (LGBTQ) in the Memphis, TN area, which is consistent with reported national rates [3]. Among these unmet needs are mental health concerns, body image concerns, and providing safe living spaces. Lifetime suicidal ideation, abuse history, higher psychological distress, and perceived discrimination based on sexuality have been reported at higher-than-average rates for black MSM and transgender women [4]. These psychosocial, structural, social network, and socio-demographic factors significantly affect HIV vulnerability among young Black MSM and transgender women and correlate with disproportionally higher rates of HIV acquisition [5]. Further, Black transwomen face higher rates of HIV risk factors, such as homelessness and increased engagement in transactional sex, than Black MSM [6]. In Tennessee (project site), 20% of respondents identifying as transgender were unemployed, 34% were living in poverty, 28% experienced housing discrimination, and 17% experienced homelessness in the past year [7]. These known social determinants of health increase personal vulnerability for HIV. It is paramount to promote overall wellness in the lives of sexual gender minorities. The eight dimensions of wellness encompass financial, occupational, intellectual, emotional, mental, physical, social, and environmental factors [8], all of which are congruent with determinants of HIV. 

In an effort to address wellness, an educational and support program for young adults was developed in collaboration with a grassroots community agency comprised of same gender loving Black men and Black transwomen. Efforts to support a community-led partnership that enhances the power and capacity of community members is supported in the literature [9]. Described herein is the process and post-evaluation findings of this health education program designed for Black sexual and gender minorities and their allies in the context of program delivery during pre- and inter-COVID-19 pandemic conditions. Community-based participatory research (CBPR) approaches [10] were used in pursuit and receipt of state funding, project conceptualization, implementation, and evaluation. The inaugural wellness program—The Memphis Headliners Whole YOUniversity (hereafter, Whole YOUniversity)—was delivered twice, once in 2019 (pre-pandemic) and again in 2020 (inter-pandemic). Implementing Whole YOUniversity during the COVID-19 pandemic required significant adaptations and provided many lessons learned to the CBPR partnership, as described in this manuscript.

## 2. Materials and Methods

### 2.1. CBPR Partnership 

In collaboration with The Memphis Headliners grassroots LGBTQ Community Advisory Group, we sought to address total wellness by attending to HIV social determinants. Collectively, we conceptualized and implemented Whole YOUniversity 1.0 and 2.0, a series of educational training sessions on culturally congruent topics for LGBTQ individuals living in the U.S. South. The longer-term goal of Whole YOUniversity was to equip the Headliners and their social/sexual networks with the capacity and skills to manage HIV, reach HIV viral suppression, or maintain HIV-negative status. Whole YOUniversity aimed to (a) improve overall wellness, (b) provide an inclusive space for social/community support to strengthen self-identity and empowerment, and (c) enhance socioeconomic stability among sexual gender minorities.

### 2.2. Participant Recruitment

The recruitment goal for each Whole YOUniversity session was 25 participants. This was a convenience sample. The CBPR partnership believed a class size of 25 was consistent with a manageable university or secondary education size classroom to facilitate both intimate small and larger group discussion. Individual interviews were conducted with a convenience sample of participants who self-selected to offer more unstructured qualitative feedback upon completing the structured evaluation form.

### 2.3. Program Procedures

Whole YOUniversity 1.0 was implemented over a 4-week period, including 3 educational training sessions addressing: (1) Sexual Health and Personal Hygiene, (2) Road to Better Career/Credit Wellness, and (3) Mental Health Hygiene. Each session was held in-person at a Memphis community-based organization serving sexual gender minority youth and the content of the trainings and speakers was informed by the Headliners group. Evidence-based didactic and experiential learning strategies were adapted to engage the priority population as suggested by the Headliners. At program conclusion, a commencement ceremony was held. Participants shared images of how the program affected them and received a completion certificate.

Whole YOUniversity 2.0 was implemented over a 5-month period, including 3 virtual education sessions addressing: (1) Body Positivity, (2) Yoga practice, and (3) Mindfulness. Sessions were held virtually using the Zoom platform. Two additional in-person training sessions occurred upon county-wide COVID-19 restrictions being lifted in May and June 2021. There was a repeat of Yoga Practice held at a public park. The fourth session covering sexual health and consent entitled “Sex Talk in the Club” took place at a popular LGBTQ night club in Memphis, TN. A comparison of participant attendance may be found in Appendix Table A1.

### 2.4. Materials and Assessment

At the end of each educational session, a self-administered post-assessment survey was given to each participant to measure satisfaction with the training, current knowledge and intentions, and areas to improve the educational experience. We also conducted brief individual interviews with participants to understand important takeaways learned from the training, how the training made the participant feel, perceived challenges applying session content, and suggestions to improve the session.

### 2.5. Data Analysis

Descriptive statistics (frequency counts) were conducted to assess post-session results. Individual interviews were recorded and transcribed verbatim. Transcripts were verified by the lead author and analyzed using a concept-driven coding approach [11].

### 2.6. Ethical Considerations

The protocol was reviewed by the University Institutional Review Board and determined non-human subjects research (PRO-FY2019-369). Participation in this program was voluntary and each volunteer provided consent and was given a copy of the informed consent for their personal records. Participants were reminded of confidentiality guidelines for group education sessions at the beginning of each session. Mental health referrals were offered when requested by participants.

## 3. Results and Lessons Learned

### 3.1. Sample Characteristics

A total of 63 participants engaged in 1 of the 3 program sessions for Whole YOUniversity 1.0. Over two-thirds (67.6%) of participants were aged 20–29 years old. While the program prioritized young adults, one-third of the sample fell into an older adult range (11.8% 30–40 years, 17.6% 40+ years). Almost a quarter of the sample reported an annual income < $10,000 (23.5%) and about half of the sample reported a range of $20,000–39,999 (47%). Almost 80% self-identified as Black, 56% same-gender loving, and 15% transgender.

In contrast, Whole YOUniversity 2.0 engaged 40 participants in 1 of the 5 sessions. The majority of participants (60%) were between the ages of 25 and 34, 20% were between 18 and 24, and 20% were 35 and older. Most participants (82.5%) self-identified as Black or African American. For gender, 42.5% of participants self-identified as cisgender men, 40% as cisgender women, and 12.5% as non-binary. There were no participants that identified as transgender. While the program prioritized gay, lesbian, and same-gender loving participants, only 40% identified as gay or lesbian, 10% as bisexual, and 7.5% as same-gender loving. All participants reported having at least some college or higher education, including 47.5% with a bachelor’s degree, 12.5% with a master’s degree, and 2.5% with a doctorate degree. The breakdown of participant characteristics may be found in Appendix Table A2.

Most participants (72.5%) reported having heard of PrEP (pre-exposure prophylaxis), a medication taken to prevent HIV, but only 25% had ever used PrEP and 22.5% reported never having come in contact with local media promoting PrEP uptake. However, 60% reported being aware of healthcare providers and community organizations that provide PrEP. Most (72.5%) considered their HIV risk low and 75% reported having ever been tested for HIV, with 42% having been tested in the last 6 months. When asked about having used at-home rapid HIV tests, 62.5% reported never having used an at-home test but 60% said they knew places to get one.

### 3.2. Program Satisfaction

In general, participants in Whole YOUniversity 1.0 and 2.0 unanimously provided positive feedback on the post-session structured evaluation form. Participants reported a high level of satisfaction with presenters (76–100% of participants rated “strongly agree” across all categories), feeling empowered (60–90% of participants), feeling included (67–100%), and feeling supported (76–85% of participants). Intentions to improve wellness in sexual health, finances, body image, and mental health/mindfulness also were reported (67–92% of participants).

At the completion of Whole YOUniversity 1.0, we hosted a commencement ceremony and a large group discussion to gather feedback on program implementation. After each session of 2.0, we met with participants individually to gather feedback. In general, we received positive responses about the speakers, format, and content. Some participants had concerns with minor aspects of the program such as sound quality, timing to ask questions, and desiring more advanced notice of events. Most of the feedback was positive. A summary of salient quotes may be found in Appendix Table A3.

The CBPR partnership reflected on lessons learned upon completion of both iterations of Whole YOUniversity 1.0 (pre-pandemic) and 2.0 (inter-pandemic). We describe five lessons learned and opportunities for growth below.

- Utility of hybrid format

Flexibility to pivot during the COVID-19 pandemic allowed the partnership to implement Whole YOUniversity 2.0. The Headliners met bi-weekly for six months in a virtual space to plan the implementation of the wellness program. Collectively, the team brainstormed session topics and identified speakers and dates for program delivery. The team used a combination of learning formats including structured PowerPoint presentations, video clips, music, small group discussion, and interactive features such as a live yoga instruction, anonymous chat box feature, and survey polls. The team took previously implemented in-person strategies to deliver health information and translated those techniques into the hybrid format. The Headliners’ partnership with the university allowed the team access to a more unrestricted Zoom account with a longer time allowance and recording capabilities. When COVID-19 restrictions were loosened, we were able to shift programming outdoors (yoga event) and indoors (Sex Talk in the Club) with proper safety precautions (e.g., smaller numbers, masks, physical spacing). While the hybrid format allowed us to deliver programming during the COVID-19 pandemic, it may not have been a feasible option for those without access to a compatible device or a private space to participate. The virtual space also did not support automatic compensation for the participants’ time (e.g., food, physical gift card) but rather necessitated verification processes and electronic gift card distribution.

- Importance of CBPR partnership

The strength of the Headliners’ partnership with other community organizations, trusted allies, and university research partners allowed for the team to successfully apply for and receive funding to support and sustain the next iteration of Whole YOUniversity during the COVID-19 pandemic. The partners were determined to respond to the feedback received in the implementation of 1.0 to add additional wellness training sessions for the implementation of 2.0. We requested support from respected and trusted allies to present to the community on topics related to their expertise for a small honorarium. The university managed the funds because the pandemic slowed the progress of the Headliners in securing 501 c 3 status. Typically, Headliner events would occur at other partners’ social and HIV service agencies to successfully reach priority populations. In lieu of in person meet-ups, we relied on the university virtual platform space to conduct the project. Recruitment was still led by the Headliners group and leveraged their social networks using social media and word of mouth recruitment.

- Innovation in virtual platform

Disruptively innovative community engagement defines the Headliners’ 5+ year partnership, as noted by previous work [12]. Foot traffic and physical community spaces facilitated recruitment goals during the delivery of 1.0. For instance, pre-pandemic partner agencies offered drop-in testing and other opportunities for clients to receive PrEP navigation and health education. During the pandemic, engagement in virtual space was met with challenges unseen in prior outreach work for the Headliners. This was the first time the Headliners relied primarily on web technology and teleconferences to engage priority groups. This is contrary to the premise of community-engaged approaches to which the Headliners typically subscribe. In this way, recruitment was more of a challenge in the virtual format. In the future, the Headliners aim to increase social media capacity for the co-chairs and place a stronger emphasis on rebranding by attending trainings by our local Ending the HIV Epidemic (EHE) coalition. The team also struggled with areas to incorporate signature and novel programs typically endorsed by the Headliners in the virtual platform. With limited options to meet in person, innovation prevailed mostly in the context of topical areas of discussing body positivity and shaming, and mindfulness to maintain one’s balance during a stressful COVID-19 pandemic. Both are unique topics and an added bonus to the menu of options to address overall wellness and social determinants of HIV. Further, these topics were seen as responsive to the current needs of the priority audience and thus served their mental, physical, and emotional state.

- Value of social media presence and upkeep

Social media presence was the main strategy to promote wellness sessions. The Headliners created flyers to advertise events and initiated Facebook Live prior to each session to garner community support. The low number of followers, views, and reactions likely contributed to the low virtual attendance. This suggested to the team the value of raising the Headliners’ visibility in other ways. For instance, the co-chairs sent direct messages to influential and respected members of the LGBTQ community to solicit interest. Team members also advertised the events using larger networks including the statewide HIV listserv, local HIV coalition distribution list, and public library information resource hub. Unfortunately, several of these attempts to broaden the scope of virtual recruitment goals attracted healthcare providers who were not the priority group. This led to restructuring future recruitment efforts by working closely with other allies with a larger social media following geared toward our intended audience. Virtual recruitment and delivery led to increases in engagement amongst LGBTQ allies. While not necessarily the intended audience for this wellness program, allies may benefit from the information and use it to further support and advocate on behalf of the priority population.

- Multi-method evaluation approaches

During the implementation of 1.0 and 2.0, all participants completed a consent form, demographic sheet, and PrEP knowledge and HIV testing behaviors assessment before the start of each training session. These assessments were intended to be used by each speaker to craft session content and weave into the overall discussion. After each session, the co-chairs reminded participants of free community rapid HIV testing and PrEP linkage services and a post-test was administered. During the pre-pandemic Whole YOUniversity 1.0 sessions, we gave each participant a printed survey and a writing utensil. However, during the pandemic, the team had to adjust administration. We created a Qualtrics survey link and emailed the link to registered participants and included the link in the chat box. Unfortunately, we had very few respondents. Given the low overall response rate, we created more interactive polls with both the pre-assessment and post-assessment questions embedded in the live Zoom, which proved to increase engagement. Additionally, knowing the community quite well and previously conducting 1.0, we knew to offer an alternative option to gather participant feedback. Brief individual interviews were conducted after each session to obtain more candid responses in real-time. This proved to be another important strategy as we issued the gift card upon completion of the interview and obtained salient feedback on the satisfaction of the session.

## 4. Discussion

Whole YOUniversity strived to address broad social determinants of HIV among Black MSM and Black transwomen. Our post-workshop evaluations for both Whole YOUniversity 1.0 (*n* = 63) and 2.0 (*n* = 40) illustrated high levels of satisfaction with selected presenters and the overall training. Participants reported feeling empowered and supported as a result of the training. Participants asked to have additional supportive resources (e.g., referrals) on hand to help facilitate their next steps.

The Headliners co-developed and co-implemented Whole YOUniversity 1.0 and 2.0, similar to other community-led partnerships aimed to develop a health promotion curriculum [9]. The value of community involvement in the design is important for the success of the program and maintenance of the program over time. Topics were informed by the community and based on immediate community need. Our priority population expressed struggling with mental health prior to the pandemic which prompted us to incorporate this track. Other research finds that Black MSM and Black transgender women reported experiencing lifetime suicidal thoughts, associated with abuse history, psychological distress, and perceived sexuality discrimination, which confirms the partnership decision [4].

Participants of Whole YOUniversity 2.0 were provided with training on how to improve one’s perception and beliefs around body image and/or body positivity. Evidence suggests that Black transgender women are at-risk for gender- and weight-related body dissatisfaction and disordered eating [13]. The Headliners knew that sexual and gender minorities engaged in Memphis needed safe spaces to discuss this issue and be supported. The data demonstrate a link between safe spaces and supportive environments and healthier outcomes among Black transwomen, particularly addressing mental health needs, reducing stress and isolation, and increasing access to health information [14].

Whole YOUniversity 1.0 relied on community partners and agencies to host wellness trainings. The Headliners were intentional about this approach to reach priority groups. In one study, Black MSM used safe spaces in community-based organizations to address the social and economic marginalization that drives HIV vulnerability [15]. Similarly, Whole YOUniversity 1.0 offered financial health training in safe spaces to build capacity skills and ultimately improve health outcomes. Basic budgeting and financial literacy may be tied to health outcomes, as one study demonstrated that the self-rated health of low-income participants was associated with measures of economic security, such as housing or food security [16].

Our wellness program was initially aimed to reach younger audiences. However, when the Headliners hosted 1.0, we quickly realized a need to be more inclusive of a wider age range. Intergenerational spaces of support can have psychological, physical, and community benefits for well-being [17]. Having engaged sexual and gender minority groups for years, the Headliners knew the importance of including mental health and mindfulness in Whole YOUniversity 1.0 and 2.0. Given the data on lifetime suicidal thoughts, associated with abuse history, psychological distress, and perceived sexuality discrimination among Black MSM and transgender women [4], it was imperative to promote a more inclusive and supportive space for intergenerational group dialogue and wellness training in person and virtually. Group-based training has been effective in addressing minority-related stress in subsets of the LGBTQ population [18]. Moreover, delivery of online LGBTQ sexual health-related information is feasible [19], as Whole YOUniversity 2.0 was able to demonstrate with the hybrid delivery of the programming.

As we continue our partnership with the Headliners, we hope to strengthen the capacity of the partners to implement and evaluate Whole YOUniversity 3.0. We are working to build the capacity of the Headliners to apply for and serve as the fiduciary entity. The Headliners have plans to file for 501 c 3 status. In 2020, the Headliners received a PoWERUp grant from Gilead COMPASS Initiative to develop their vision and mission statements. We hope to better equip the group with skills in Culturally Responsible and Equitable Education (CREE) and dissemination [20]. We currently have a book chapter in production and have already submitted conference abstracts and delivered presentations to build research capacity. As researchers, we are also building our capacity in measurement and cultural sensitivity to strengthen future iterations of Whole YOUniversity.

### Limitations

The major limitation of the program is that we were not scaled to deliver a randomized controlled trial to assess efficacy. This was beyond the scope of the work at this time. This funding mechanism allowed for us to pilot a program to obtain preliminary data to inform the development of a more rigorously evaluated program in the future. Further, the two iterations of Whole YOUniversity collected demographic data differently, with the first allowing more flexibility in participant responding and the second having more discrete demographic options. This change was made to more accurately capture participant demographics and will be used in further iterations in the future.

## 5. Conclusions

The recruitment goal of 25 participants was reached in 2 of 3 sessions in the implementation of Whole YOUniversity 1.0. Participants requested additional supportive resources (e.g., onsite job fairs, referral resources, mental and social support) to help facilitate their next steps, which prompted the partnership to apply for and secure additional funds for Whole YOUniversity 2.0. The Headliners took the feedback from earlier participants to include program topics on body positivity and mindfulness. One primary next step as we re-emerge and recover from the COVID-19 pandemic is to use some of the best practices presented here to integrate the Headliners’ signature program into existing LGBTQ advisory group activities. Future programs will broaden the reach to a larger sample of youth as well as use social media and rebrand innovatively to attract more participants and engage a larger audience in meaningful ways.

## Data Availability

Access to data collected for the study will be limited to members of the project team.

## Appendix A

**Table A1:** Comparison of attendance for Whole YOUniversity 1.0 (pre-COVID) vs. 2.0 (during COVID).

Scheme	Topic	pre-COVID (*n*)	Topic	During COVID (*n*)
1	Sexual Health Hygiene	11 ^a^	Body Positivity *	3
2	Financial Health Hygiene	25	Mental Health (yoga) *	7
3	Mental Health Hygiene	27	Mental Health (mindfulness) *	3
4	---	---	Yoga in the Park	15
5	---	---	Sex Talk in the Club	12
Total		63		40

* Virtual. ^a^ Severe weather impacted attendance and reaching the goal of 25 participants.

**Table A2:** Participant characteristics for Whole YOUniversity 1.0 (pre-COVID) vs. 2.0 (during COVID).

	Pre-COVID (*n* = 34)	During COVID (*n* = 40)
Age		
18–24	11	8
25–34	15	24
35–44	1	4
45–60	6	4
Missing	1	0
Income ^a^		
<$10,000	8	---
$10,000–19,999	4	---
$20,000–29,999	8	---
$30,000–39,999	9	---
>$40,000	4	---
Missing	1	---
Education		
High School Graduate	7	0
Some college/trade	11	7
2-year degree/Associate degree	1	5
4-year degree/Bachelor’s degree	10	19
Graduate or professional degree	5	6
Prefer not to specify education	---	1
Missing	0	2
Self-identification *		
Same gender loving	19	3
Gay, lesbian, queer, homosexual	---	16
Bisexual, pansexual	---	4
Transgender	5	0
Heterosexual/straight	---	2
Other sexuality	---	12
Prefer not to specify sexuality	---	3
		
Black	27	33
White	---	3
Multiracial	---	2
Other race/ethnicity	---	2 ^b^
		
Cis Male	---	17
Cis Female	---	16
Non-binary, gender fluid, non-conforming	---	5
Other gender	---	1
Prefer not to specify gender	---	1
Other open-ended self-identification	3 ^c^	0
Missing	0	0
County of Residence		
Shelby	31	35
Other County	3	3
Not sure	---	2
Missing	0	0

^*^ Participants were allowed to check all applicable categories. ^a^ Did not assess income during COVID. ^b^ Did not specify ‘other race/ethnicity’, ^c^ Christian *n* = 1; Queer *n* = 1; African American, Latino mix, and SGL *n* = 1.

**Table A3:** Post-session feedback from Whole YOUniversity participants.

Lesson Learned	Representative Participant Quote
Utility of hybrid format	-There was some dead space in between but that’s because of general lag. This is a new way of communication due to the current pandemic and for us being safe. -Allow the audience to ask more questions. -We as individuals can’t take that for granted because, in a day like today, you never know who you’re getting. Instagram, Facebook, and all this facade. When you need a real person, then they say that they’re who they are. -… I’m wanting to get back into the community. So I was game for whoever may be here, whether they’re old friends, new friends, or just complete randoms.
2. Importance of CBPR partnership	-Before I came here, I felt very comfortable because knowing the people that I’ve been associated with throughout the years of Headliners and then new people and what you all bring to the table, I felt very confident as far as a safe space. -Thank you for giving us, and providing the space where we can truly just be ourselves and explain how we feel without any judgment and all that. -This was an awesome program, and I think people definitely want this back again and for a longer period! I can’t wait to work on the next project!
3. Innovation in virtual platform	-Because if you come to the Zoom, you’re meeting and people that you wouldn’t normally see or talk to. You guys make it a really comfortable place for people to feel a part of the situation. -An hour is plenty of time. I feel in today’s world, you were lucky to have gotten people to be there for an hour. -The talk was good. The communication was good. The facilitators were awesome.
4. Value of social media presence and upkeep	-I guess by promoting it ahead of time, like, better timing
5. Multi-method evaluation approaches	-So, like I said, if there were more people involved with it, you know, we probably could have like been able to take conversations deeper or further so that, you know, because of more robust conversation versus just asking questions and responding to questions.
